# Association of the human platelet antigens polymorphisms with platelet count in patients with COVID-19

**DOI:** 10.3389/fmed.2023.1265568

**Published:** 2023-11-06

**Authors:** Kazem Ghaffari, Mahsa Ashrafi Rad, Amin Moradi Hasan-Abad, Mersedeh Khosravi, Arefeh Benvidi, Mahsa Iraji, Heidar Ali Heidari Khargh, Ali Ghasemi

**Affiliations:** ^1^Student Research Committee, Khomein University of Medical Sciences, Khomein, Iran; ^2^Department of Laboratory Sciences, Khomein University of Medical Sciences, Khomein, Iran; ^3^Department of Biochemistry and Hematology, Faculty of Medicine, Semnan University of Medical Sciences, Semnan, Iran; ^4^Autoimmune Diseases Research Center, Shahid Beheshti Hospital, Kashan University of Medical Sciences, Kashan, Iran; ^5^Department of Biochemistry, Semnan University of Medical Sciences, Semnan, Iran; ^6^Cancer Research Center, Semnan University of Medical Sciences, Semnan, Iran

**Keywords:** coronavirus disease, thrombocytopenia, human platelet antigen polymorphisms, platelet count, PCR

## Abstract

Polymorphism in human platelet antigen (HPA)-1 and HPA-3 (GPIIb/IIIa), HPA-2 (GPIb/IX), HPA-4 (GPIIIa), HPA-5 (GPIa/IIa), & HPA-15 (CD109) was investigated in 86 COVID-19-infected patients with thrombocytopenia (Group A) and 136 COVID-19-infected patients without thrombocytopenia (Group B). HPA genotyping was done by the sequence-specific primers PCR method. Lower HPA-3a and higher HPA-3b (*P* = 0.028) allele frequencies were seen in Group A than in Group B, and homozygosity for HPA 3b (*P* = 0.038) alleles was more prevalent in Group A than in Group B. The allele and genotype distributions of the other HPA polymorphic variants were similar between the two groups. Univariate analysis identified the CCGGGC (*P* = 0.016) combined genotype to be negatively associated & the TCGGGC (*P* = 0.003) and CCGGGC (*P* = 0.003) to be positively associated with thrombocytopenia. The frequency of anti-HPA-1a and anti-HPA-3a antibodies was significantly higher in all patients compared to other anti-HPAs antibodies (*P* < 0.05). These results highlight the role of HPAs in the thrombocytopenia of COVID-19 infected patients. This is the first evidence demonstrating the differential association of the six common HPA gene variants and specific HPA genotype combinations with thrombocytopenia in COVID-19-infected patients.

## Introduction

New coronavirus pneumonia is a new human respiratory disease developed by severe acute respiratory syndrome coronavirus 2 (SARS-CoV-2), officially named Coronavirus 2019 (COVID-19) by the World Health Organization (1). Severe patients often develop shortness of breath with or without hypoxemia, which eventually progresses to acute respiratory distress syndrome, septic shock, coagulation dysfunction, incurable metabolic acidosis, and multiple organ failure (2). Patients with COVID-19 have similar hematologic changes as seen in patients with Middle East Respiratory Syndrome (MERS) (3). Thrombocytopenia and lymphopenia are common blood disorders in patients with COVID-19, indicating poor patient survival (4). The prevalence of thrombocytopenia in COVID-19 patients is highly variable (2). The exact causes of thrombocytopenia in patients with COVID-19 remain unknown, but some events such as increased platelet (PLT) destruction, reduced PLT production, and increased PLT consumption may lead to thrombocytopenia in patients with COVID-19 (5). PLT membrane glycoproteins express several antigenic features on their surface that have a polymorphism, known as human PLT antigens (HPAs) (6). To date, forty-one HPAs have been described in the Immuno Polymorphism Database (IPD)^1^ (7). Among the types of HPAs, variants of HPA-1 to -5 and -15 are considered due to their high prevalence and clinical importance (8–11). HPAs have polymorphisms in populations and most of these polymorphisms are caused by the replacement of an amino acid in the structure of a protein by the replacement of a nucleotide in the structure of deoxyribonucleic acid (DNA) (12, 13). Association between some polymorphisms of the HPA system with some diseases such as post-transfusion purpura (PTP), fetal-neonatal alloimmune thrombocytopenia (NAIT) (14), myocardial infarction (15), stroke (16), venous thrombosis (17) and the progression of liver fibrosis (18) have been shown in some studies. The distribution of these gene variants is geographically and ethnically restricted (19, 20).

Based on a literature search, this is the first study that shows the association between polymorphisms of HPA-1 to -5 and -15 and the PLT count in patients with COVID-19, making this study unprecedented.

## Materials and methods

### Study population and laboratory methods

Two hundred and twenty-two patients with newly diagnosed COVID-19 who were referred to the Khordad Hospital, Varamin, Iran, were randomly included in the study. Informed consent was obtained from all patients who participated in this study. Patients were divided into group A; 86 COVID-19-infected patients with thrombocytopenia, and in group B, 136 COVID-19-infected patients without thrombocytopenia. The normal count of PLTs is 150–450 × 10^3^ per mm^3^. Thrombocytopenia is defined as a PLT count below 150 × 10^3^/mm^3^ (21). Host and viral factors evaluated included gender, weight, age, age at infection, duration of infection, viral load, aspartate aminotransferase (AST), alanine aminotransferase (ALT), total bilirubin, prothrombin time (PT), PLT count, AST-to-PLT ratio index (APRI = [AST/upper limit of normal] × [100/PLTs, × 10^9^/L]), albumin, fast blood sugar (FBS), triglycerides (TG), cholesterol & body mass index [(BMI); weight/height squared (kg/m2)]. Demographic and clinical findings were obtained from patients’ records. We confirmed the diagnosis of COVID-19 via reverse transcriptase-polymerase chain reaction (RT-PCR) assays performed on nasopharyngeal swab specimens.

Inclusion criteria were: COVID-19 infection confirmed by serological testing and molecular assays. Exclusion criteria were as follow: acute and chronic liver diseases, fibrosis, cirrhosis, thrombotic thrombocytopenic purpura patients, heparin-induced thrombocytopenia, infections (such as hepatitis B virus, human immunodeficiency viruses, hepatitis C virus or coinfections), history of autoimmune disease, hypertension, splenomegaly/hypersplenism, pregnancy-related thrombocytopenia, gastrointestinal bleeding, cancer, transfusion of blood products, severe iron deficiency anemia, aplastic anemia, intravenous injection of human immunoglobulin, taking anti-PLT drugs such as aspirin, corticosteroids, and other diseases that cause thrombocytopenia. This study was approved by the Ethics Committee of Semnan University of Medical Sciences (Ethical committee code number: IR.SEMUMS.REC.1401.046). All experiments were performed by relevant guidelines and regulations.

### DNA extraction

Five ml of patients’ peripheral blood samples were collected on admission in sterile tubes containing EDTA anticoagulant. DNA extraction was performed using the Brasilica kit (LGC Biotecnologia, Brazil) kit using the standard manufacturer’s protocol. Briefly, 200 μl of lysis solution was added to 200 μl of the patient’s blood sample, and after adding 20 μl of proteinase K, it was placed at 60°C for 15 min. Then 100 μl of isopropanol was added and the solution was transferred to a DNA extraction column. After centrifugation, washing solutions 1 and 2 were added, and after the final centrifugation, 100 microliters of Elution buffer were added, and after centrifugation, DNA was extracted. DNA concentration was determined spectrophotometrically at 260 nm (A260) absorption using NanoDrop1000 (Thermo Scientific).

### Sequence-specific primers PCR

Genotyping of the 6 HPA polymorphisms [HPA-1 T196C, HPA-2 T524C, HPA-3 T2622G, HPA-4 G526A, HPA-5 G1648A & HPA-15 A2108C (22)] was performed by Sequence-Specific Primers PCR (PCR–SSP), as described previously (23) with slight modifications. Briefly, to perform the PCR–SSP assay, the kit was used in such a way that 10 μl of Red master mix amplicon (Taq polymerase 2x and Mgcl_2_ 1.5 μM) and 1 μl of forward primer, 1 μl of reverse primer, 4 μl of water and 4 μl of DNA Template were mixed and vortexed. Two sets of primers, each comprising an allele-specific and a common primer, were employed for the recognition of each allele. Negative control was carried out in all PCR runs.

### Detection of PLTs associated antibodies

The frequency of target HPAs of anti-PLT antibodies in thrombocytopenic patients was assessed by Quantitative monoclonal immobilization of PLT antibodies (MAIPA). Since the frequencies of different HPA genes vary in different populations, MAIPA for different IgG antibodies against HPA-1, -2, -3, -4, -5, and -15 is needed. HPA-1, HPA-3, and HPA-4 are present on the GPIIb/IIIa, while HPA-2 is found on the GPIb/IX, HPA-5 is carried on the GPIa/IIa, and HPA-15 is localized to CD109 (22). MAIPA assay is an enzyme-linked immunosorbent assay (ELISA) technique for the detection and identification of HPA antibodies. The MAIPA assay was performed as described previously (24). In short, human PLT-Antibody Screening Cells and PLT-Antibody Identification Panel Cells (apDiaMed AG Kit, Switzerland) were incubated with the plasma of patients with COVID-19 and washed three times with phosphate-buffered saline (PBS) containing 0.05% EDTA. By adding PBS containing 1% Triton X-100, soluble PLT lysate was prepared and added to the pre-coated 96-well microtiter plates with Goat Anti-Mouse IgG Fc fragment specific (Jackson ImmunoResearch, West Grove, PA). After 1 hour (h) incubation, antibodies in the plasma of COVID-19 patients bound to HPA were detected with the final antibody (horseradish peroxidase-conjugated goat-antihuman IgG Fc fragment specific) and a substrate solution containing 3,3’,5,5’-tetramethylbenzidine. The reaction is stopped by adding H_2_SO_4_. The absorbance of the plate was read at 450 nm on an automated microtiter plate reader. The optical density (OD) values above 0.2 were considered positive.

### Statistical analysis

Pearson’s χ2 test or Fisher’s exact test was utilized to assess associations between the genotypic frequency of the HPA-1 to -5 and -15 systems with PLT count. Also, Pearson’s χ2 test (or Fisher’s exact test) and Student’s *t*-test were utilized for qualitative variables and quantitative variables, respectively. Deviation from Hardy-Weinberg equilibrium was analyzed by Pearson’s χ2 test. The multivariate logistic regression model was carried out to determine an independent association of HPA genotypes with thrombocytopenia. HPA combined genotype estimation was performed by the maximum-likelihood procedure. HPA genotype combinations were coded as per the allele (wild type or mutant) at each locus. The first letter refers to HPA-1 (T allele = 1a, C allele = 1b), the second to HPA-2 (T allele = 2a, C allele = 2b), the third to HPA-3 (T allele = 3a, G allele = 3b), the fourth to HPA-4 (G allele = 4a, A allele = 4b), the fifth to HPA-5 (G allele = 5a, A allele = 5b) and the sixth to HPA-15 (A allele = 15a, C allele = 15b). Odds ratios (OR) and 95% confidence intervals (CI) were determined by Univariate analysis. Statistical analysis was performed with the SPSS version 24.0 software (SPSS Inc., Chicago, IL, USA) and a genetic analyzer (ABI PRISM 310, Applied Biosystems). Statistical significance was set at a 0.05 level for all the tests.

## Results

### Demographic characteristics

There were a total of 222 patients admitted with a median age of 41.6 years old, ranging from 24 to 69 years. A total of 124 patients (55.8%) were male, and 98 (44.2%) were female. The demographic and clinical data of all the patients included are demonstrated in Table 1. No statistically significant differences were found between the patient groups about age, gender, BMI, AST, ALT, albumin, viral load, FBS, cholesterol, triglyceride, PT, total bilirubin, creatinine, LDH, WBCs, absolute lymphocyte count, and PLT-to-lymphocyte ratio. APRI, D-dimer, death rate, and duration of hospitalization were significantly higher in thrombocytopenic SARS-CoV-2 positive patients compared with non-thrombocytopenic patients (*P* < 0.05).

**TABLE 1:** Clinical characteristics of patients at admission.

Variable	Thrombocytopenic SARS-CoV-2 positive (*n* = 86)	Non-thrombocytopenic SARS-CoV-2 positive (*n* = 136)	*p*-value
Age, years [median (IQR)]	38.5 (27–69)	41.1 (24–65)	0.207
Gender (M/F)	45/41	79/57	0.409
Body weight (kg)*	76.1 ± 7.8	77.4 ± 4.9	0.142
Body mass index (*kg/m2*)*	32.7 ± 3.4	33.7 ± 2.4	0.118
Aspartate transaminase, U/l (IQR)	41.3 (16–83)	43.5 (21–89)	0.190
Alanine transaminase, U/L (IQR)	34.9 (7–129)	41.6 (11–165)	0.201
Platelet count, ×10^3^/μL*	110.4 ± 48.8	262.2 ± 53.6	**0.012**
AST-to-platelet ratio index (IQR)	0.49 (0.20–1.14)	0.44 (0.14–1.11)	**0.001**
Albumin, g/d*	4.2 ± 0.95	4.3 ± 0.93	0.846
SARS-CoV-2 RNA, UI/ml*	1238889.6 ± 1663703.3	1553931.2 ± 1979994.6	0.258
Fast blood sugar *	106.5 ± 17.4	110.6 ± 39.6	0.219
Cholesterol, mg/dl*	149.7 ± 34.7	150.1 ± 40.8	0.824
Triglyceride, mg/dl*	102 ± 31. 6	103.2 ± 33	0.826
Prothrombin time (s) (IQR)	12.1 (10–15)	12.2 (10–13)	0.967
Total bilirubin, mg/dl*	0.25 ± 0.16	0.28 ± 0.17	0.278
Creatinine, μmol/L	75.3 (62.4–89.8)	71.6 (60.5–86.7)	0.358
D-dimer, μg/mL	0.7 (0.5–6.2)	0.3 (0.1–1.4)	**0.021**
Death rate, *n* (%)	11 (12.8)	7 (5.2)	**0.042**
Lactate dehydrogenase	438.6 ± 231.4	394.6 ± 224.8	0.354
White blood cells (×10^9^/L)	7.8 ± 1.9	8.1 ± 2.4	0.598
Absolute lymphocyte count (×10^9^/L)	1.8 ± 2.7	2.1 ± 1.1	0.231
Platelet-to-lymphocyte ratio*	115.3 ± 125.1	231.7 ± 88.4	0.01
Time of hospitalization*, days	11.3 ± 5.2	8.6 ± 4.3	**0.03**

For quantitative variables, data are provided as the median (IQR) or, if marked with *, as the mean ± standard deviation. SARS-CoV-2 RNA: severe acute respiratory syndrome coronavirus 2 ribonucleic acid; n, number; IQR, interquartile range. Bold indicates *p* < 0.05.

### Genotype and allele frequencies of HPAs

The HPA-1 to -5 and -15 genotypes and allele frequencies in patients with SARS-CoV-2 infection are summarized in Table 2. There were no significant differences in the genotype and allele frequency distribution for the HPA-1, -2, -4, -5, and -15 between thrombocytopenic SARS-CoV-2 positive patients and non-thrombocytopenic patients (*P* > 0.05). However, distributions of the genotype and allele frequency for HPA-3 were significantly different between the two groups. The allele frequency of HPA-3b [G allele] in the thrombocytopenic patients was found to be significantly higher than in the non-thrombocytopenic patients, and HPA-3a [T allele] in the thrombocytopenic patients was significantly lower than in the non-thrombocytopenic patients (*P* = 0.028). The genotype frequency of HPA-3bb [2622 G/G] was significantly higher in thrombocytopenic patients than that in non-thrombocytopenic patients, and the genotype frequency of HPA-3aa/ab [2622 T/T & T/G] was significantly lower in thrombocytopenic patients than that in the non-thrombocytopenic patients (*P* = 0.038). The OR of the HPA-3bb [2622 G/G] for thrombocytopenia was 3.425 (95% CI 1.275–9.199), and the OR of the HPA-3b [G allele] was 1.617 (95% CI 1.051–2.488). Genotypes did not deviate from the Hardy–Weinberg equilibrium in the HPA systems and revealed no indication of linkage disequilibrium (Table 3). Multivariate logistic regression showed an independent association between the genotype HPA-3aa (*P* = 0.014; OR [95% CI], 5.666 [1.412–22.737]) and HPA-3ab (*P* = 0.011; OR [95% CI], 5.827 [1.504–22.575]) with thrombocytopenia, when compared with genotype HPA-3bb, independent of the age at infection (*P* = 0.720) (Table 4).

**TABLE 2:** HPA allele and genotype frequency.

HPAs	Allele/Genotype	Thrombocytopenic SARS-CoV-2 positive (*n* = 86)	Non-thrombocytopenic SARS-CoV-2 positive (*n* = 136)	*p*-value*	OR (95% CI)
	T allele	165 (95.9)	257 (94.5)	0.494	1
	C allele	7 (4.1)	15 (5.5)		0.727 (0.290–1.821)
HPA-1	T/T	79 (91.9)	121 (89)	0.483	1
	T/C	7 (8.1)	15 (11)		0.715 (0.279–1.831)
	C/C	0	0		
	T allele	122 (70.9)	205 (75.4)	0.301	1
	C allele	50 (29.1)	67 (24.6)		1.254 (0.816–1.927)
HPA-2	T/T	44 (51.2)	75 (55.1)	0.339	1
	T/C	34 (39.5)	55 (40.5)		1.054 (0.598–1.857)
	C/C	8 (9.3)	6 (4.4)		2.273 (0.740–6.980)
	T allele	118 (68.6)	212 (77.9)	**0.028**	1
	G allele	54 (31.4)	60 (22.1)		1.617 (1.051–2.488)
HPA-3	T/T	45 (52.3)	83 (61)	**0.038**	1
	T/G	28 (32.5)	46 (33.8)		1.123 (0.620–2.033)
	G/G	13 (15.2)	7 (5.2)		3.425 (1.275–9.199)
	G allele	172 (100)	272 (100)	1.000	
	A allele	0	0		
HPA-4	G/G	136 (100)	136 (100)	1.000	–
	G/A	0	0		
	A/A	0	0		
	G allele	172 (100)	272 (100)	1.000	
	A allele	0	0		
HPA-5	G/G	136 (100)	136 (100)	1.000	–
	G/A	0	0		
	A/A	0	0		
HPA-15	A allele	73 (42.4)	137 (50.4)	0.103	1
	C allele	99 (57.6)	135 (49.6)		1.376 (0.937–2.022)
	A/A	11 (12.7)	32 (23.6)	0.136	1
	A/C	51 (59.3)	73 (53.6)		2.032 (0.938–4.402)
	C/C	24 (28)	31 (22.8)		2.252 (0.946–5.365)

*Pearson’s χ^2^ test. Number (percent of total). Bold indicates *p* < 0.05.

**TABLE 3:** The evaluation of the distribution of gene frequencies of HPA-1, HPA-2, HPA-3, and HPA-15 by Hardy–Weinberg equilibrium test.

Genotypes		Thrombocytopenic SARS-CoV-2 positive			Non-thrombocytopenic SARS-CoV-2 positive		
		EN	ON	*p*-value	EN	ON	*p*-value
HPA-1	T/T	79.1	79		121.4	121	
	T/C	6.7	7	0.793	14.2	15	0.925
	C/C	0.1	0		0.4	0	
	T/T	43.3	44		77.3	75	
HPA-2	T/C	35.5	34	0.582	50.5	55	0.929
	C/C	7.3	8		8.3	6	
	T/T	40.5	45		82.6	83	
HPA-3	T/G	37.0	28	0.981	46.8	46	0.076
	G/G	8.5	13		6.6	7	
	A/A	15.5	11		34.5	32	
HPA-15	A/C	42.0	51	0.691	68.0	73	0.140
	C/C	28.5	24		33.5	31	

ON, observed number; EN, expected number.

**TABLE 4:** Multivariate regression logistic of risk factors related to thrombocytopenia.

Variable			
**HPA-3: T/T vs. G/G**			
HPA-3: T/G vs. G/G	2.54	**0.011**	5.827 (1.504–22.575)
Age at infection	−0.35	0.720	0.982 (0.891–1.083)

Bold indicates *p* < 0.05.

### HPA genotype combinations

Analysis of the six-locus HPA combined genotypes is shown in Table 5. Of the 62 HPA genotype combinations recognized, selected HPA genotype combinations were associated with thrombocytopenia. These included the TCGGGC (*P* = 0.001) & TTGGGC (*P* = 0.029) combined genotypes, which were higher, and the CCGGGC (*P* = 0.001) & TCTGGC (*P* = 0.047) combined genotypes, which were lower among thrombocytopenia patients compared to non-thrombocytopenic patients. Univariate analysis identified the CCGGGC (*P* = 0.016) combined genotype to be negatively associated & the TCGGGC (*P* = 0.003) and CCGGGC (*P* = 0.003) to be positively associated with thrombocytopenia (Table 6).

**TABLE 5:** HPA combined genotypes in thrombocytopenic and non-thrombocytopenic SARS-CoV-2 positive patients.

Combined genotype*	Thrombocytopenic SARS-CoV-2 positive (%)	Non-thrombocytopenic SARS-CoV-2 positive (%)	*p*-value
TTTGGA	73 (42.2)	137 (50.4)	0.119
TTTGGC	45 (26.2)	68 (25)	0.823
TCTGGC	0	7 (2.6)	**0.047**
TCGGGC	50 (29.1)	45 (16.5)	**0.001**
CCTGGC	0	15 (5.5)	**0.001**
TTGGGC	4 (2.3)	0	**0.029**

*HPA combined genotype (HPA-1, to -5, and -15) frequency determined by the maximum likelihood method. Fisher’s exact test. Bold indicates *p* < 0.05.

**TABLE 6:** Distribution of HPA combined genotypes.

	Univariate		
Combined genotype*	Z score	*P*	OR (95%CI)
TTTGGA			1.00 (reference)
TTTGGC	0.847	0.397	1.242 (0.775–1.991)
TCTGGC	1.911	**0.056**	1.051 (1.013–1.091)
TCGGGC	2.967	**0.003**	0.480 (0.293–0.785)
CCTGGC	−2.967	**0.003**	1.109 (1.053–1.169)
TTGGGC	2.408	**0.016**	0.948 (0.900–0.999)

*HPA combined genotype (HPA-1, to -5, and -15) frequency determined by the maximum likelihood method. Combined genotypes were coded according to the allele (wild type or mutant) at each locus. Bold indicates *p* < 0.05.

### Evaluation of PLT count

**FIGURE 1:**
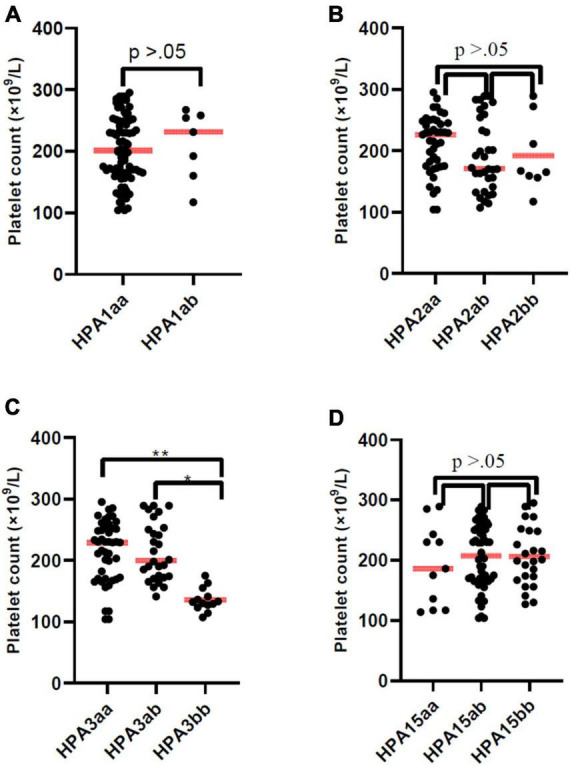
Platelet count of patients according to HPA-1, -2, -3, and -15 genotype. The line through the middle of the box is the median. Platelet count of patients with HPA-3bb genotype was significantly lower than patients with genotype HPA-3aa or HPA-3ab (*P* < 0.05).

**FIGURE 2:**
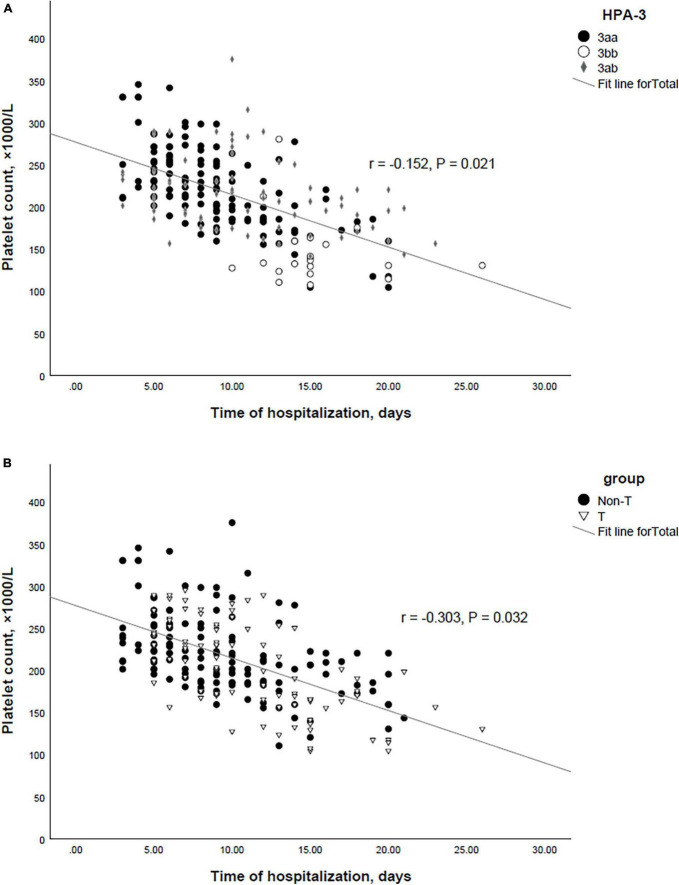
**(A)** Correlation between the HPA-3 and time of hospitalization in SARS-CoV-2 positive patients. **(B)** Correlation between the platelet count and time of hospitalization in SARS-CoV-2 positive patients.

The mean PLT counts ± SD was significantly lower in thrombocytopenic SARS-CoV-2 positive patients compared to non-thrombocytopenic patients (110.4 ± 48.8 vs. 220.4 ± 48.8, × 10^3^/μL; *P* = 0.012). The mean PLT counts did not differ among HPA-1, -2, -4, -5, and -15 genotypes (Figures 1A, B, D). However, the mean PLT counts of patients with the HPA-3bb genotype was significantly lower than patients with genotype HPA-3aa/ab (*P* < 0.05) (Figure 1C). In thrombocytopenic SARS-CoV-2 positive patients, 71.2% with HPA-3bb genotype had a low PLT count compared with 32.3% of those with HPA3aa/ab genotype. In non-thrombocytopenic patients, 57.4% with the HPA-3bb genotype had a low PLT count compared to only 27.6% of those with the HPA-3aa/ab genotype, using the normal cut-off of 150,000/μL. The mean ± SD time of hospitalization was 9.6 ± 4.8 days. Time of hospitalization was significantly and negatively correlated with the observed PLT count in two groups (Figure 2A). Also, correlation analysis showed that hospitalization time was significantly and negatively correlated with patients’ PLTs based on HPA-3 genotype (Figure 2B). The duration of hospitalization was 8.5 ± 4.3 for HPA-3aa, 11.7 ± 5.1 for HPA-3ab, and 15.9 ± 3.5 for HPA-3bb.

### MAlPA results in patients

**FIGURE 3:**
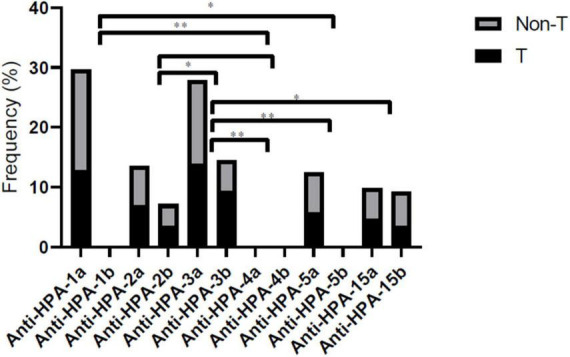
Comprehensive distribution of platelet-specific antibodies according to HPA-1, -2, -3, -4, -5, and -15 genotype in thrombocytopenic/non-thrombocytopenic SARS-CoV-2 positive. Statistical significance was set at a 0.05 level for all the tests. *P < 0.05, and ^**^P < 0.01. T, thrombocytopenic; Non-T, non-thrombocytopenic.

Until now, the MAIPA assay was the specific method for PLT autoantibody detection. Thus we performed autoantibody detection in patient samples using the MAIPA method (25). In SARS-CoV-2 positive patients, MAIPA was positive in 54 of 222 (24.3%) (23/86 thrombocytopenic patients and 31/186 non-thrombocytopenic patients). The highest frequency of target HPAs of anti-PLT antibodies in thrombocytopenic patients was HPA-1a (12.7%), HPA-2a (6.9%), HPA-2b (3.4%), HPA-3a (13.9%), HPA-3b (9.3%), HPA-5a (5.8%), HPA-15a (4.6%), HPA-15b (3.4%), and all cases negative for HPA-1b, HPA-4a, and HPA-4b (Figure 3). The highest frequency of target HPAs of anti-PLT antibodies in non-thrombocytopenic patients was HPA-1a (16.9%), HPA-2a (6.6%), HPA-2b (3.6%), HPA-3a (13.9%), HPA-3b (5.1%), HPA-5a (6.6%), HPA-15a (5.1%), HPA-15b (5.8%), and all cases negative for HPA-1b, HPA-4a, and HPA-4b (Figure 3). The frequency of anti-HPA-1a and anti-HPA-3a antibodies was significantly higher in all patients compared to other anti-HPAs antibodies (*P* < 0.05).

## Discussion

In this study, thrombocytopenia occurred in 38.7% of patients with COVID-19. In line with these findings, a study in China that included 1099 patients showed that 36.2% of patients had thrombocytopenia (26). In total, it has been determined that 5-41% of COVID-19 patients suffer from thrombocytopenia (27). Also, previous studies have shown that patients with severe COVID-19 disease present with thrombocytopenia and lymphopenia more than those with non-severe disease. These patients were more likely to develop acute respiratory distress syndrome (28, 29).

Numerous studies have tried to determine the relationship between the severity of COVID-19 disease and the rate of disease progression by examining different variables to use the results along with other factors in diagnosing and determining the prognosis of patients and also managing treatment. Therefore, we studied the PLT count changes in patients with COVID-19 and the association between thrombocytopenia and the polymorphisms of HPA-1 to -5 and -15. To our knowledge, this is the first study to evaluate the association of HPA polymorphisms with PLT count in patients with COVID-19 infection.

Our study showed that in patients with COVID-19, the HPA-3bb genotype and HPA-3b allele are associated with a low PLT count. This study also identified HPA genotypic combinations associated with thrombocytopenia in COVID-19 patients. A significant finding was the reduction of PLT counts in TCGGGC and TTGGGC genotypes. While the combined genotypes TCTGGC and CCTGGC were not associated with a decrease in PLT count.

The HPA polymorphisms are related to a wide variety of clinical conditions (17, 18) and their pattern of distribution is affected by ethnicity (23, 30). Several previous studies have reported a possible association between the susceptibility to hepatitis C virus (HCV) infection and the gene frequencies of HPAs (18, 31). The association between HPA-1 polymorphic variants and fibrosis progression was previously reported by Silva et al. (31) in Brazilian HCV-infected patients.

The HPA-3 (T2622G, Ile843Ser) is present on the human PLT GPIIb. GPIIb is the alpha subunit of the PLT integrin GPIIb-IIIa receptor complex. The GPIIb/IIIa receptor complex performs various functions, including interaction with the endothelium and a role in the risk of myocardial infarction and thrombosis (12, 32–34). On the other hand, Hamaia et al. (35) reported that PLTs can act as carriers of HCV. There is not enough information about the direct interaction of HCV and COVID-19 with PLT. However, integrins such as glycoprotein or other adhesion molecules can play an important role in the interaction of viruses such as HCV or COVID-19 with PLTs.

The pathogenesis of thrombocytopenia in patients with COVID-19 is not fully understood. Severe changes in the immune response and autoimmunity have been reported in some viral infections, including HCV (36). From previous studies, it is known that antibodies against PLT surface antigens in patients with HCV can cause immunological destruction of PLTs (37). In a study about the role of immune mechanisms as a causative agent of thrombocytopenia in HCV hepatic patients, the authors found that PLT-associated GP antibodies cause thrombocytopenia in patients with chronic HCV infections. Also, Their results revealed that the highest frequency of target HPAs of anti-PLT antibodies in thrombocytopenic HCV-positive hepatic patients was HPA-1 (46.7%), HPA-2 (10%), HPA-3 (46.7%), HPA-4 (6.7%), HPA-5 (10%) and all cases negative for HPA-15 (38). These findings were to some extent similar to the results of this study. We detected PLT-specific antibodies in the patient’s plasma to ensure a more accurate and credible conclusion of the relationship between PLT-specific antibodies and thrombocytopenia. These findings suggest an immune-based mechanism of thrombocytopenia in COVID-19-infected patients.

Moreover, we found a significant negative correlation between PLT count and time of hospitalization in SARS-CoV-2-positive patients. The average hospitalization day of patients with thrombocytopenia was longer than those without thrombocytopenia. In line with this finding, one study reported that patients with a higher platelet count had shorter hospitalization than patients with a lower platelet count at the time of admission (29).

Therefore, several hypotheses that lead to thrombocytopenia in patients with COVID-19 can be expressed as follows:

(a) Under normal physiological conditions, PLTs do not adhere to the vascular endothelium unless the PLT-endothelium interaction is disrupted (39, 40). The attachment of COVID-19 to platelets through HPA may cause the adhesion of platelets to the endothelium of blood vessels and subsequently thrombocytopenia by disrupting the PLT-endothelium interaction.
(b) In clinical trials and preclinical studies, it has been determined that GPIIb/IIIa receptor antagonists are effective inhibitors of platelet aggregation so that these antagonists reduce platelet half-life (41). The binding of COVID-19 to the GPIIb/IIIa receptor through HPA may cause the virus to act as an antagonist of this receptor and subsequently cause thrombocytopenia.
(c) PLT degradation may be due to the presence of anti-PLT antibodies with or without the presence of immune complexes that react with specific glycoproteins or HPAs.
(d) Binding of COVID-19 to PLTs via HPAs may lead to the formation of neo-antigens on the PLT surface, resulting in the formation and production of antibodies against target HPAs. In addition, it can be assumed that the immune complexes attached to the PLT surface may play an important role in PLT destruction, possibly through phagocytosis by macrophages of the reticuloendothelial system. Therefore, HPA polymorphisms may play a role in the immune-mediated clearance of platelets in the reticuloendothelial system of patients with COVID-19.

To confirm these hypotheses, it is suggested to evaluate the glycoproteins of PLT levels and PLT-specific antibodies in patients with COVID-19.

## Conclusion

These results highlight the role of HPAs in the thrombocytopenia of COVID-19-infected patients. Also, PLT-specific antibodies represent a common mechanism inducing thrombocytopenia in COVID-19-infected patients. We presented the first evidence suggesting the distinct association of specific HPA genotype combinations with thrombocytopenia in COVID-19-infected patients. Considering the population and ethnic heterogeneity in the distribution of HPA polymorphisms and their possible pathogenic capacity, it is recommended to evaluate the role of HPA polymorphisms as risk factors for thrombocytopenia in different populations with COVID-19.

## Data availability statement

The original contributions presented in this study are included in the article/supplementary material, further inquiries can be directed to the corresponding author.

## Ethics statement

The studies involving humans were approved by the Ethics Committee of Semnan University of Medical Sciences (Ethical Committee code number: IR.SEMUMS.REC.1401.046). The studies were conducted in accordance with the local legislation and institutional requirements. Written informed consent for participation in this study was provided by the participants’ legal guardians/next of kin.

## Author contributions

KG: Conceptualization, Writing—original draft. MR: Data curation, Investigation, Writing—original draft. MK: Funding acquisition, Software, Writing—original draft. AB: Data curation, Resources, Writing—original draft. MI: Resources, Software, Writing—original draft. HK: Software, Writing—original draft, Data curation. AG: Conceptualization, Project administration, Supervision, Validation, Visualization, Writing—review & editing.

## Footnotes

1
https://www.versiti.org/hpa
